# Oxcarbazepine Induced Rhabdomyolysis and Acute Kidney Injury: A Case Report

**DOI:** 10.1155/crin/4168888

**Published:** 2025-05-16

**Authors:** Sara Yavuz Ileri, Nuray Can Usta

**Affiliations:** ^1^Nephrology Department, Dr. Abdurrahman Yurtaslan Oncology Training and Research Hospital, Ankara, Türkiye; ^2^Neurology Department, Health Science University, Trabzon Kanuni Training and Research Hospital, Trabzon, Türkiye

**Keywords:** acute kidney injury, oxcarbazepine, rhabdomyolysis

## Abstract

The cause of acute skeletal muscle damage, defined as rhabdomyolysis, is multifactorial. Medicines are prominent among the most important reasons. The clinical significance of rhabdomyolysis lies in the fact that it can cause acute kidney injury (AKI) in 10%–40% of patients. In this presented case, oxcarbazepine, which is a preferred drug in the treatment of epilepsy and also in neuropathic pain in clinical practice, triggered rhabdomyolysis and subsequently caused the development of AKI. Through this case report, we aimed to emphasize that some serious side effects should not be forgotten when prescribing drugs in clinical practice.

## 1. Introduction

Rhabdomyolysis or acute skeletal muscle damage may develop due to various etiologies. Trauma, excessive physical exercise, immobilization, hypophosphatemia, infections, hypokalemia, some autoimmune diseases, hypo- or hyperthyroidism, hypothermia, and hyperthermia, especially opioids, antiepileptics, statins, and alcohol, can cause rhabdomyolysis, which leads acute kidney injury (AKI) in 10%–40% of patients [1]. The main cause of AKI is the direct toxic effect of myoglobulin. In pathogenesis, iron ions play a significant role because of tubular obstruction. After rhabdomyolysis, multiple organ failure may occur due to the high amounts of substances such as potassium, lactic acid, and myoglobin in the circulation. This syndrome, called AKI due to rhabdomyolysis (RIAKI), is unfortunately a common complication that affects 46% of the patients requiring hospitalization and 80% of the patients requiring intensive care [2, 3]. Oxcarbazepine, a 10-keto derivative of carbamazepine, is a first- or second-line treatment for primary generalized tonic-clonic seizures and focal-onset epilepsy [4]. It is recommended to use it in the treatment of neuropathic pain such as diabetic neuropathy and radiculopathy in some studies but there is not much evidence for its effectiveness.

In this case, we aimed to present an AKI case that occurred because of rhabdomyolysis due to oxcarbazepine treatment for trigeminal neuralgia. This is the first case in literature in which the rhabdomyolysis side effect is presented [5]. We believe that it is important to remember this rhabdomyolysis effect of oxcarbazepine, which is more preferred than carbamazepine in the clinic by virtue of its safer side effects.

## 2. Case Presentation

A 70-year-old female patient presented to the clinic with the complaint of pain that affected her right side of her face. Her history contained diabetes mellitus, hypertension, bronchial asthma, and hyperthyroidism. At admission, her physical and neurological examinations were normal. Laboratory studies showed slightly elevated blood glucose only. The patient was diagnosed with trigeminal neuralgia and treated with oxcarbazepine 150 mg twice daily. On the third day of the treatment, drowsiness was observed. The patient reported decreased red or brown urine. Her serum creatinine, myoglobin and creatine phosphokinase levels were 4.0 mg/dL, 1000 ng/mL, and 765 U/L, respectively. Urine myoglobin level was 1700 ng/mL.

According to laboratory results and clinical presentation, acute renal failure caused by rhabdomyolysis was diagnosed and oxcarbazepine treatment was discontinued. The patient was referred to the intensive care unit immediately. Intravenous hydration and supportive care were implemented. In the follow-up, it was noted that the apathy state of the patient was mended, serum creatinine levels had decreased to 1.2 mg/dL, and urine output and color improved. Therefore, the patient was referred to the internal medicine inpatient clinic because of the recovery of AKI. After the creatinine value returned to the basal level, the patient was discharged for follow-up at the nephrology outpatient clinic [6]. Laboratory tests performed along with timeline are displayed in Table 1.

## 3. Discussion

Trigeminal neuralgia caused by demyelination of the cranial nerves is a sharp, intermittent, and superficial pain that follows the nerve trace. Anticonvulsants are the first preferred group of drugs. Oxcarbazepine is one of the most preferred anticonvulsants in clinical practice with a safe side-effect profile.

While carbamazepine is more commonly associated with side effects such as dizziness, hyponatremia, rash, and hepatic dysfunction, one of the most important among them is rhabdomyolysis, which is also observed in other anticonvulsants. But the list of drugs which exert elevated risk for rhabdomyolysis is prolix. HMG-CoA reductase inhibitors, a tyrosine kinase inhibitor (i.e., sunitinib), DNA-binding anticancer drugs (i.e., trabectedin), disease-modifying antirheumatic drug (i.e., leflunomide), and short acting anesthetics (i.e., propofol) are best known [7].

The mechanism is not fully understood but may involve direct myotoxicity, drug interactions increasing plasma levels, hypersensitivity reactions, hyponatremia-induced muscle injury, and genetic susceptibility in certain individuals.

Uncontrolled diabetes can also lead to rhabdomyolysis due to electrolyte imbalances, dehydration, and muscle ischemia, which can be developed during the diabetes mellitus. Peripheral vascular diseases and neuropathy are the main prominent factors which can facilitate the process. Some medications which are commonly used in treatment such as SGLT2 inhibitors could contribute by increasing dehydration situations additionally.

Although rare, hyperthyroidism can induce rhabdomyolysis even though it is more commonly associated with thyrotoxicosis, especially in severe or untreated hyperthyroidism due to muscle breakdown. Increased metabolic demand, muscle overuse, and secondary effects like hypokalemia are the most accelerating triggers for rhabdomyolysis.

The diagnostic laboratory test for rhabdomyolysis is the measurement of plasma creatine kinase. In general, a level greater than 10,000 IU/L confirms severe rhabdomyolysis and is considered a high risk for the development of RIAKI. In this case, RIAKI was diagnosed based on elevated serum myoglobin (1000 ng/mL), creatine phosphokinase (765 U/L), and creatinine (4.0 mg/dL).

Oxcarbazepine, a keto derivative of carbamazepine, has similar efficacy as carbamazepine in the treatment of epilepsy. In addition, its tolerance is high, and its side effect profile is better. It is a good alternative in the treatment of trigeminal neuralgia [8].

However, some of the side effects have high clinical significance. One of them is hyponatremia. It is the most reported side effect. Therefore, fluid restriction is recommended along with the use of the drug in clinical practice. Hyponatremia is a serious and important side effect because it can increase the frequency of seizures.

Rhabdomyolysis is also one of the important side effects of oxcarbazepine. However, despite being stated as a side effect profile, we have not encountered any clinically defined cases of rhabdomyolysis in the literature [9].

For this reason, the major limitation of this case is that it is based on a single patient which restricts the generalizability of the findings to broader populations. The absence of additional supporting literature or similar reported cases limits the ability to establish a clear association between oxcarbazepine and rhabdomyolysis. Without a larger dataset or corroborative evidence, it is difficult to determine whether the observed condition represents a rare but real clinical phenomenon or an isolated incident. This highlights the need for further case reporting to better understand the potential link and underlying mechanism between two conditions.

While oxcarbazepine is generally well tolerated, clinicians should remain vigilant for early signs of muscle toxicity such as unexplained muscle pain, weakness, or dark-colored urine. Routine monitoring of creatine kinase levels may be considered especially in patients with additional risk factors such as diabetes or hyperthyroidism.

Although based on a single case, the clinical applicability of this report lies in raising awareness of a potential adverse effect, encouraging more cautious prescribing practices and prompting timely investigation when symptoms arise.

Hence, we aimed to recall this side effect of this new anticonvulsant, which is more preferred than carbamazepine in epilepsy, bipolar disorders, and trigeminal neuralgia. Further studies are needed to establish evidence-based monitoring protocols and to better understand the incidence and mechanism of oxcarbazepine-induced rhabdomyolysis.

## Figures and Tables

**Table 1:** Timeline and laboratory findings.

Day	Clinical event/observation	Laboratory test	Result	Reference range
Day 0	Initiation of oxcarbazepine for trigeminal neuralgia	Serum creatinine	1.1 mg/dL	0.6–1.2 mg/dL
		Fasting blood glucose	135 mg/dL (7.5 mmol/L)	70–100 mg/dL (3.9–5.6 mmol/L)
		TSH	4.5 mU/L	0.45–4.12 mU/L
		Blood urea nitrogen (BUN)	18 mg/dL	
		White blood cells (WBCs)	6.400/mm^3^	4500–11,000/mm^3^
		Red blood cells (RBCs)	4.8 million/mm^3^	Male: 4.3–5.9 million/mm^3^ Female: 3.5–5.5 million/mm^3^
		Hematocrit (HT)	15 gr/dL	Male: 41%–53% Female: 36%–46%
		Mean corpuscular volume (MCV)	95 μm^3^	80–100 μm^3^
		Mean corpuscular hemoglobin (MCH)	32 pg/cell	25.4–34.6 pg/cell
		Platelets	350.000/mm^3^	150,000–400,000/mm^3^
		Potassium	4.20 mEq/L	3.5–5.0 mEq/L
		Aspartate aminotransferase (AST)	22 U/L	10–40 U/L
		Alanine aminotransferase (ALT)	18 U/L	10–40 U/L
		Albumin	4.2 g/dL	3.5–5.5 g/dL

Day 3	Patient reports fatigue, mild muscle pain	Serum sodium	136 mmol/L	135–145 mmol/L
	Onset of dark-colored urine, muscle weakness	Creatine phosphokinase (CPK)	765 U/L	22–198 U/L
		Serum myoglobin	1000 ng/mL	< 70 ng/mL
		Serum creatinine	4.0 mg/dL	0.6–1.2 mg/dL
		Blood urea nitrogen (BUN)	48 mg/dL	7–20 mg/dL

Day 4	Discontinuation of oxcarbazepine	—	—	—

Day 5	Supportive treatment initiated	Serum sodium	138 mmol/L	135–145 mmol/L

Day 7	Improvement in symptoms	Creatine phosphokinase (CPK)	260 U/L	22–198 U/L
		Serum creatinine	2.3 mg/dL	0.6–1.2 mg/dL

Day 10	Urine output and color improved	Serum creatinine	1.2 mg/dL	0.6–1.2 mg/dL

## Data Availability

The data used to support the findings of this study are available from the corresponding author upon reasonable request.
